# 794 Use of Cultured Epithelial Autografts After Biodegradable Temporizing Matrix in Massive Burns

**DOI:** 10.1093/jbcr/irac012.344

**Published:** 2022-03-23

**Authors:** Jason Heard, David L Wallace, Soman Sen, Tina L Palmieri, David G Greenhalgh, Kathleen S Skipton Romanowski

**Affiliations:** University of California - Davis, Sacramento, California; University of California, Davis, Sacramento, California; UC Davis, Sacramento, California; UC Davis and Shriners Hospitals for Children Northern California, Sacramento, California; University of California - Davis, Sacramento, California; UC Davis, Sacramento, California

## Abstract

**Introduction:**

As burn care advances, patients are surviving with larger burn injuries, that previously would have been fatal. However, the need for autologous skin coverage continues to be an unmet need for massive burn injuries. Several attempts have been made to address this with various dermal substitutes, temporary coverage, and skin substitutes. For 25 years, Cultured Epithelial Autografts (CEA) have been used to treat large burn injuries, but this was met with variable success and has a mandatory pre-requisite lab time before it is ready for use. In 2018, Biodegradable Temporizing Matrix (BTM) that can be placed immediately on excised burns was first studied in burn patients, which has led to its increased use in subsequent years. This case series seeks to examine our experience using CEA following the application and ingrafting of BTM on large burns.

**Methods:**

A retrospective review was conducted from 2017-2020 of adult burn patients admitted to an ABA verified burn center who underwent placement of both BTM and CEA. Demographics, mechanism of injury, burn characteristics, surgeries, and outcome data were collected. Surgical technique was early excision, BTM placement, a BTM integration period, repeat superficial excision, fibrin/thrombin spray, split thickness skin grafting with usually 6:1 mesh autograft, and finally CEA application. CEA was managed per manufacturer protocols. Descriptive statistics and univariate analyses were performed with Microsoft Excel.

**Results:**

Eight patients met inclusion criteria. The average age was 29.3±5.3 years, 2nd degree TBSA 22.5±22.6%, 3rd degree TBSA 55.8±21%, and total TBSA was 78.3±4.4%. Four patients died during their hospital course and four survived to discharge. For survivors, the age length of stay was 135±23.6 days and they underwent an average of 8.5±1.5 total excision and/or grafting procedures. All patients had severe complications including severe sepsis/septic shock (n=8), gastrointestinal bleeds (n=2), acute respiratory distress syndrome (n=3), acute kidney injury or renal failure (n=4), pulmonary embolism (n=1) and myocardial infarction (n=1). The average time to 95% wound closure was 5 (79-147) days for survivors.

**Conclusions:**

There continues to be an unmet need for autologous skin coverage in massive burn injuries when there is insufficient donor skin. In this series, we describe eight patients with massive burn injuries who underwent initial BTM placement, followed by 6:1 meshed autograft and CEA application. Although four patients died during their treatment course, the four surviving patients had acceptable wound closure rates and length of stay for their burn size.

